# Acknowledgement to Referees

**DOI:** 10.1186/s40798-016-0070-z

**Published:** 2017-01-06

**Authors:** Steve McMillan, Roger Olney

**Affiliations:** Springer Healthcare, 5 The Warehouse Way, Northcote, Auckland New Zealand

Dear Reader,

As we approach the end of 2016, we would like to thank all those who have contributed to *Sports Medicine - Open* over the past 12 months.

Firstly, we must acknowledge how much we are indebted to all of the authors who have submitted articles to *Sports Medicine - Open* over the course of 2016. Their skill and dedication, along with their support for this journal, are critical to the journal’s success.

The quality of the articles published is also testament to the significant efforts of the peer reviewers, whose commitment ensures that the journal’s content is held to the highest possible standard. We would like to thank the following individuals who, in addition to members of the Honorary Editorial Board, acted as reviewers for articles published in *Sports Medicine - Open* over the last 12 months:

**Maicon Albuquerque**

Brazil

**Maurice Arnaud**

Switzerland

**Patricia Aronson**

USA

**Guilherme Artioli**

Brazil

**Greg Atkinson**

UK

**Todd Backes**

USA

**Seung-Soo Baek**

Republic of Korea

**Tyson Beach**

Canada

**Clint Bellenger**

Australia

**Stephen Bird**

Australia

**Barbara Bowsher**

USA

**Michel Brink**

the Netherlands

**Siacia Broos**

Belgium

**Roland Büchter**

Germany

**David Buzas**

USA

**Lilian Calderón-Garcidueñas**

USA

**Karina Rabello Casali**

Brazil

**Cristina Casals**

Spain

**Ricardo Cassilhas**

Brazil

**Helmi Chaabène**

Tunisia

**Hamdi Chtourou**

Tunisia

**Francis Conidi**

USA

**Stephen Cornish**

Canada

**Robert Defraites**

USA

**Kevin Deighton**

UK

**Dominic Dellweg**

Germany

**David Docherty**

Canada

**Kenji Doma**

Australia

**Sladjana Dronjak-Čučaković**

Serbia

**Meghan Edwards**

USA

**Matthew Ely**

USA

**Damian Farrow**

Australia

**Peta Forsyth**

Australia

**Paul Freeman**

UK

**Victor Froelicher**

USA

**Thomas Gee**

UK

**Bruno Grassi**

Italy

**Tom Hazell**

Canada

**Genevieve Healy**

Australia

**Martin Hoffman**

USA

**Sharmin Hossain**

Canada

**John Hough**

UK

**Erin Howden**

Australia

**Chun-Jung Huang**

USA

**Jackie Hudson**

USA

**Florentino Huertas**

Spain

**Charlotte Huppertz**

the Netherlands

**Stefanie Hüttermann**

Germany

**Shigeki Izumi**

Japan

**Michael Jaffee**

USA

**Saša ŠegaJazbec**

Slovenia

**Bruce Jones**

USA

**Kelly Jones**

New Zealand

**Mariana Kaiseler**

UK

**Kristof Kipp**

USA

**Thomas Kjeld**

Denmark

**Beat Knechtle**

Switzerland

**Karsten Koehler**

USA

**Sylvain Laborde**

Germany

**Steen Larsen**

Denmark

**David Lavallee**

UK

**Trent Lawton**

New Zealand

**John Leddy**

USA

**Seth Lenetsky**

New Zealand

**Duncan Macfarlane**

Hong Kong

**Karin Magnussen**

Denmark

**Yorgi Mavros**

Australia

**Scott McIntosh**

USA

**Terry McMorris**

UK

**Romain Meeusen**

Belgium

**Régis Mestriner**

Brazil

**Geoffrey Minett**

Australia

**Lluís Mont**

Spain

**Colleen Muñoz**

USA

**Marius Myrstad**

Norway

**Tamas Nagy**

Hungary

**Fabio Nakamura**

Brazil

**David Nieman**

USA

**Onni Niemelä**

Finland

**Vahur Ööpik**

Estonia

**Sushil Pant**

Nepal

**Camille Perchoux**

France

**Noel Perkins**

USA

**Neal Pollock**

USA

**Denis Prud'homme**

Canada

**Joseph Purita**

USA

**Claudio Rafols Del Canto**

Chile

**Anette Ranhoff**

Norway

**Ulrike Rimmele**

Switzerland

**Rodrigo Rodrigues**

Brazil

**Kelly Russell**

Canada

**Bryan Saunders**

Brazil

**B. Volker Scheer**

USA

**Marc Scheltinga**

the Netherlands

**Alison Schinkel-Ivy**

Canada

**Stephen Simons**

USA

**Dean Simonton**

USA

**Jonathan Singer**

Canada

**Neil Smart**

Australia

**Jostein Steene-Johannessen**

Norway

**Urs Stöcker**

Switzerland

**Mogammad Sharhidd Taliep**

South Africa

**Marcus Taylor**

USA

**Matthew Tucker**

USA

**Luc van Loon**

the Netherlands

**Will Vickery**

UK

**Henry Williford**

USA

**Peter Wilmshurst**

UK

**Carl Woods**

Australia

**Léder Xavier**

Brazil

**Wanghong Xu**

China

**Julia Zakrzewski**

UK

Finally, we would like to offer our sincere thanks to the members of the journal’s Honorary Editorial Board, who have acted as peer reviewers and authors, and have provided guidance on journal content, policy and processes:

**Duarte Araújo**

Portugal

**Peter Arnett**

USA

**Sam Blacker**

UK

**Fadi Charchar**

Australia

**Jia Yi Chow**

Singapore

**Matias Costa-Paz**

Argentina

**Keith Davids**

UK

**Joel Gagnier**

USA

**Ruben Goebel**

Qatar

**John Hawley**

Australia

**Martin Hägglund**

Sweden

**Patria Hume**

New Zealand

**Mikel Izquierdo**

Spain

**Steve Kamper**

Australia

**Justin Keogh**

Australia

**Tom Loney**

United Arab Emirates

**Tim Meyer**

Germany

**Carles Pedret**

Spain

**Luis Prato**

USA

**Michael Schwenk**

Germany

**Alison September**

South Africa

**Sanjay Sharma**

UK

**Roy Shephard**

Canada

**Roberto Simão**

Brazil

**Keith Tolfrey**

UK

**Willem van Mechelen**

the Netherlands

**Mounir Zok**

USA

We hope that you have found the articles published throughout 2016 to be both interesting and informative. We have appreciated the high quality of content contributed to the journal this year and we look forward to keeping you up to date with sport and exercise science research in the year to come.

With best wishes,

Steve McMillan & Roger Olney

Editors

